# Assessment of Rates of Child Maltreatment in States With Medicaid Expansion vs States Without Medicaid Expansion

**DOI:** 10.1001/jamanetworkopen.2019.5529

**Published:** 2019-06-14

**Authors:** Emily C. B. Brown, Michelle M. Garrison, Hao Bao, Pingping Qu, Carole Jenny, Ali Rowhani-Rahbar

**Affiliations:** 1 Seattle Children’s Research Institute, Center for Child Health, Behavior, and Development, Seattle, Washington; 2 Department of Pediatrics, University of Washington School of Medicine, Seattle; 3 Division of Child Psychiatry, University of Washington School of Medicine, Seattle; 4 Department of Epidemiology, University of Washington School of Public Health, Seattle; 5 Harborview Injury Prevention & Research Center, University of Washington, Seattle

## Abstract

**Importance:**

Physical abuse and neglect affect a significant number of children in the United States. The 2014 Medicaid expansion, in which several states opted to expand their Medicaid programs, is associated with parental financial stability and access to mental health care.

**Objective:**

To determine whether Medicaid expansion is associated with changes in physical abuse and neglect rates.

**Design, Setting, and Participants:**

This ecological study used state-level National Child Abuse and Neglect Data Systems (NCANDS) data from January 1, 2010, through December 31, 2016, to compare the change in physical abuse and neglect rates in states that chose to expand Medicaid vs those that did not. All cases of physical abuse and neglect of children younger than 6 years during the study period that were referred to state-level Child Protective Services and screened in for further intervention after having met a maltreatment risk threshold were included. Cases with only documented sexual or emotional abuse were excluded. A difference-in-difference analysis was conducted from April 12, 2018, through March 26, 2019.

**Exposures:**

State-level Medicaid expansion status.

**Main Outcomes and Measures:**

Incidence rate of screened-in referrals for physical abuse or neglect per 100 000 children younger than 6 years per year by state.

**Results:**

Data were analyzed for 31 states and the District of Columbia that expanded Medicaid and 19 states that did not during the study period, with baseline neglect counts of 646 463 and 388 265, respectively. After Medicaid expansion, 422 fewer cases of neglect per 100 000 children younger than 6 years (95% CI, −753 to −91) were reported each year after adjusting for confounders for comparison of postexpansion and preexpansion rates in states that expanded Medicaid contrasting with the change during that time in nonexpansion states. From 2013 to 2016, Medicaid coverage for adults with dependent children increased a median 1.9% (interquartile range, 0.4% to 4.3%) in the states that did not expand Medicaid and 4.2% (interquartile range, 0.9% to 6.0%) in the states that did. No associations were found between Medicaid coverage or Medicaid eligibility criteria and physical abuse or neglect rates.

**Conclusions and Relevance:**

Medicaid expansion was associated with a reduction in the reported child neglect rate, but not the physical abuse rate. These findings suggest that expanding Medicaid may help prevent child neglect.

## Key Points

**Question:**

Is the state expansion of Medicaid associated with rates of child physical abuse and neglect?

**Findings:**

In this ecological study comparing pre– and post–Medicaid expansion state-level rates of child physical abuse and neglect from the National Child Abuse and Neglect Data Systems, after adjusting for confounders, there were fewer cases of reported neglect (422 fewer per 100 000 younger than 6 years) in states that expanded Medicaid than during that time in nonexpansion states, which had a baseline rate of 3944 cases per 100 000 children younger than 6 years in 2013.

**Meaning:**

These results suggest that Medicaid expansion may serve as a means to prevent child neglect.

## Teaser

This ecological study evaluates whether US Medicaid expansion was associated with state-level changes in physical abuse and neglect rates among children younger than 6 years compared with states that opted out of Medicaid expansion.

## Introduction

Multiple studies have shown that individuals who experience childhood adverse events are at greater risk of developing several diseases in adulthood, including heart disease and cancer.^1,2,3^ Five of the 10 events included on the current Adverse Childhood Experience screening questionnaire developed from these studies relate to child maltreatment, a broad term encompassing physical, sexual, and emotional abuse and neglect.^4^

The estimated total monetary cost of confirmed child maltreatment cases occurring in a single year in the United States, including lifetime medical care and productivity losses, is $124 billion to $428 billion.^5,6,7^ The lifetime cost per person who experiences maltreatment is $210 012, more than $43 000 of which represents direct medical costs.^7^ These numbers only account for confirmed cases. Child Protective Services (CPS) receives more than 3 million referrals annually, representing about 5% of all children.^8^ Once unreported cases are added, an estimated 15.2% of children in the United States experience caregiver maltreatment each year.^9^

Much work has been done to develop programs to reduce child maltreatment’s significant burden on children, families, and society. Few programs, however, have been found to be consistently effective at reducing maltreatment rates.^10,11,12,13^ Policies and programs addressing some of the risk factors for maltreatment (eg, poverty, limited parental access to mental health care) have been proposed as a means to prevent child maltreatment indirectly.^14^ One study^15^ demonstrated that California’s 2004 paid family leave policy was associated with a significant reduction in the rate of abusive head trauma compared with states without similar leave policies. Another study^16^ showed that child care access and continuity of child health care policies were also associated with decreased child maltreatment rates.

**Figure 1.**
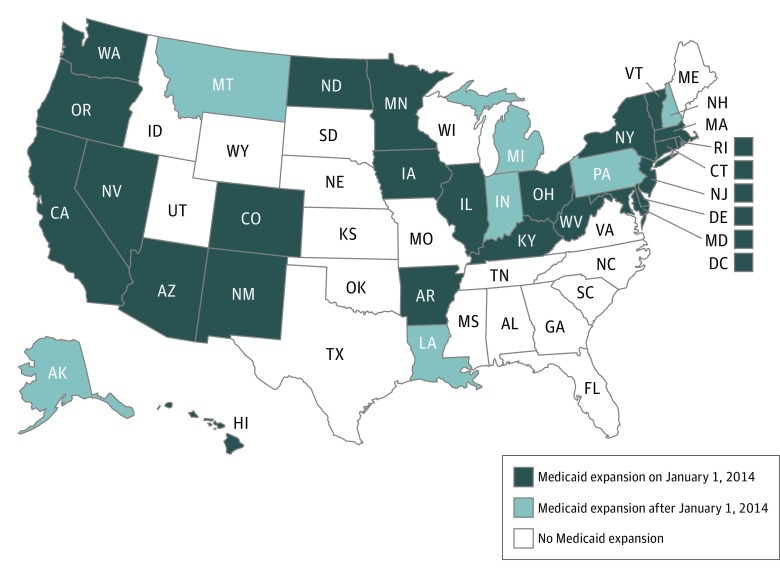
Medicaid Expansion Status by State as of December 31, 2016

On January 1, 2014, 24 states and the District of Columbia expanded their Medicaid programs to include all adults with an annual income to 138% of the federal poverty level (FPL) as part of the Affordable Care Act (ACA) (Figure 1).^17,18^ Seven other states expanded their programs at a later date, through July 1, 2016.^19^ Although the expansion was intended to roll out nationwide, the US Supreme Court determined that states could opt out, and 19 states did.^20^ The Medicaid expansion was credited in 2015 with expanding coverage to 11 million adults.^21^ This increase in medical insurance coverage had a positive effect on financial stability and several health outcomes in the lower-income population.^22,23,24,25^ The proportion of uninsured parents living in states with Medicaid expansions dropped 33% from 2013 to 2014.^26^ Studies have shown that the ACA Medicaid expansion resulted in improved mental health status for low-income parents.^22,25^

The findings from these studies suggest that expanding Medicaid was associated with parents’ financial stability and access to mental health care. Because these are important risk factors for maltreatment,^27^ Medicaid expansion may have also been associated with child maltreatment outcomes, including physical abuse and neglect. Because Medicaid expansion did not happen in all states or at the same time, this allows for a comparison not just of maltreatment outcomes within states that expanded Medicaid before and after the expansion occurred, but also the same maltreatment outcomes between states that expanded Medicaid and those that did not.

We conducted this ecological study to examine and quantify the association between state Medicaid expansion and the rates of child abuse and neglect. We hypothesized that states and years with expanded Medicaid would have lower rates of child physical abuse and neglect than would states and years without Medicaid expansion.

## Methods

We conducted an ecological study because the exposure of interest (ie, Medicaid expansion) was inherently at the group level. The goal was not to understand individual-level associations but rather the associations of a policy shift at the state level. Moreover, decisions by policy makers regarding Medicaid expansion, at least in the context of this investigation, would likely revolve around changes in aggregated state-level outcomes rather than changes for specific individuals, so the adopted design is most appropriate. The study followed the Strengthening the Reporting of Observational Studies in Epidemiology (STROBE) reporting guideline. This study was exempt from full review by the Seattle Children’s Hospital and the University of Washington institutional review boards because data were aggregated and no identifiable information was recorded; deidentification was not indicated.

### Data and Sample

We used demographic and maltreatment data from the National Child Abuse and Neglect Data System (NCANDS) Child Files (eMethods in the Supplement).^28^ This administrative data set includes child-level data for all child maltreatment reports investigated by CPS in all 50 states and the District of Columbia. We included data for 2010 through 2016 to capture trends in maltreatment rates for several years before and after Medicaid expansion. The year 2016 was used as the study cutoff because this was the most recent year for which complete data were available for the exposure, outcomes, and covariates as described below.

### Exposure

The exposure of interest was whether a state expanded Medicaid on or after January 1, 2014. State-level exposure status was determined based on Medicaid expansion information available through the Henry J. Kaiser Family Foundation (KFF).^17^ The KFF Medicaid expansion data have been used previously in other studies evaluating expansion outcomes by state.^19,24^ Information about the income eligibility for Medicaid for parents of dependent children expressed as a percentage cutoff of the FPL in each state and for each year was obtained from the KFF.^29^ States expanding Medicaid after January 1, 2014, were only included in the expanded group for the period in which they actually had expanded Medicaid.

### Outcome

The primary outcome using NCANDS data was the incidence rate of screened-in referrals for physical abuse or neglect per 100 000 children younger than 6 years per year in each state. These referrals made to CPS have met criteria at intake, indicating that an investigation is warranted for 1 or several types of maltreatment. Once an investigation is complete, the concern for maltreatment may be categorized as substantiated, unsubstantiated, or several disposition categories between. If several children are included in 1 referral, each child counts as a separate case. If the same child is referred to CPS more than once per year, that child is counted once for each referral to CPS. We used the Centers for Disease Control and Prevention Wide-ranging Online Data for Epidemiologic Research population estimates to determine the child population by state and year.^30^

We included all screened-in referrals instead of only substantiated cases. This decision was based on prior research demonstrating that the decision process for substantiation is inconsistent and subject to policy changes and biases^31^ and that little difference in parental recidivism and other outcomes seems apparent between children whose cases were substantiated and those whose cases were not.^32,33,34,35^ This decision is consistent with other recent studies using CPS records.^16,36,37,38,39^ The screened-in referrals were stratified by maltreatment type (physical abuse or neglect) and included cases screened-in for an alternative response. Cases with concern for both physical abuse and neglect were included in both groups.

Although some of the risk factors for sexual abuse are the same as for physical abuse or neglect,^27^ we chose to exclude cases in which sexual abuse was the only maltreatment type; perpetrators in cases of sexual abuse are less likely to be parents or guardians than in cases of physical abuse or neglect, so Medicaid expansion was not thought to affect these cases in the same way, even if the perpetrators were eligible for Medicaid through the expansion.^40,41,42^ We also excluded cases in which maltreatment type or disposition was missing or emotional abuse was the only maltreatment type, because this category is inconsistently captured in CPS records owing to the absence of a consensus definition,^43,44^ because some states do not include emotional abuse as a separate category of maltreatment,^45^ and because studies have shown that emotional abuse is significantly underreported. Maltreatment deaths were also excluded because state identifier data are masked in NCANDS for cases resulting in death such that we were unable to determine their exposure status.

### Intermediate Outcome

We used KFF state-level data on the estimated number of adults with dependent children who have insurance coverage to determine the association of Medicaid expansion policies with Medicaid coverage among parents in each state, a possible intermediate step in the association between Medicaid expansion policies and child maltreatment outcomes.^46^ The KFF estimates are based on additional analyses of the yearly US Census Bureau’s Current Population Survey^47^ and the Annual Social and Economic Supplements.^48^

### Covariates

We included the following state-level variables as possible confounders based on factors shown to be associated with the outcomes of physical abuse and neglect and thought to be associated with the exposure, Medicaid expansion. Paid family leave policy status was determined from the National Partnership for Women & Families data.^49^ Unemployment proportion was obtained from the US Bureau of Labor Statistics.^50^ Teenage birth rate data were obtained from the National Center for Health Statistics National Vital Statistics System.^51^ The percentage of families with children younger than 5 years living below the FPL was determined using the American Community Survey estimates from the US Census Bureau.^52^ Finally, child care wait list data were obtained from the National Women’s Law Center.^53^

### Statistical Analysis

We compared prespecified baseline characteristics of expansion and nonexpansion states in 2013. To compare the change in physical abuse and neglect reporting rates in states that expanded Medicaid vs those that did not during the 7-year period of interest, we conducted difference-in-difference analyses with general linear regression models with state and year as fixed effects (eMethods in the Supplement) in which the regression coefficient for Medicaid expansion is interpretable as a generalized form of difference-in-difference.^54^ Difference-in-difference analyses allow for the comparison of a change in mean outcome between a group that has experienced a treatment and a group that has not experienced the treatment before and after that treatment occurs.

We calculated descriptive statistics, including median and interquartile range (IQR) for the change in Medicaid eligibility. A general linear regression model with state and year as fixed effects was used to examine the association between the Medicaid expansion exposure and the change in Medicaid eligibility that was treated as a continuous outcome. To further analyze the association between the intermediate outcome of change in Medicaid eligibility and the final outcomes of interest, physical abuse and neglect, general linear regression analyses were performed. The same analyses were conducted for the change in parental Medicaid coverage.

For all the models, to identify influential data points, the difference-in-fits approach was used.^55^ This diagnostic technique omits each observation, one at a time, and refits the regression model to determine whether particular observations have an outsized influence on the fitted model. This approach identified an unexplained near doubling of West Virginia’s physical abuse rates, possibly due to a CPS intake change, in 2014.^56^ West Virginia was therefore excluded from analyses that included physical abuse or neglect outcomes, although secondary analyses including West Virginia were conducted to evaluate the exclusion’s potential impact. Analyses were also conducted excluding several other potential outliers. The number of averted cases after expansion was calculated based on the estimate for the outcome rate and the known child population in the nonexpansion states for the years 2014 through 2016.

All analyses were conducted using SAS, version 9.4 (SAS Institute, Inc). The analyses were conducted from April 12, 2018, through March 26, 2019. All 2-sided *P* values were considered statistically significant at *P* < .05.

## Results

We analyzed NCANDS data for 31 states and the District of Columbia that expanded Medicaid and 19 states that did not during the period from January 1, 2010, through December 31, 2016. Table 1 shows that before Medicaid expansion (in 2013), the median teen birth rate and the proportion of families with children younger than 5 years living below the FPL were higher in the states that did not expand Medicaid than in the states that did. Conversely, the proportion of parents with Medicaid coverage was lower in the states that did not go on to expand Medicaid.

**Table 1.** Comparison of the States With vs Without Medicaid Expansion in 2013

Baseline Characteristic	States^a^	
	With Medicaid Expansion (n = 31)^b^	No Medicaid Expansion (n = 20)^c^
No. of children aged <6 y by state	279 291 (79 339-535 097)	354 489 (198 220-550 928)
Total No. of children aged <6 y	14 008 018	9 845 257
Unemployment rate, %	7.3 (6.6-8.1)	6.7 (5.3-7.4)
Teen birth rate per 1000 women	23.6 (17.7-30.3)	29.6 (24.8-34.5)
US population below FPL, %^d^	16.8 (13.5-21.2)	20.7 (18.3-23.6)
Parents with Medicaid coverage, %	14.6 (12.3-19.5)	9.2 (8.0-13.8)
States with childcare wait list, No. (%)	12 (39)	7 (35)
States with paid leave, No. (%)	2 (6)	0

Abbreviation: FPL, federal poverty level.

^a^ Unless otherwise indicated, data are expressed as median (interquartile range).

^b^ Includes Washington, DC.

^c^ Louisiana began expansion in July 2016 and is included among the states that did not expand Medicaid.

^d^ Includes parents with children younger than 5 years.

From 2013 to 2016, Medicaid coverage for adults with dependent children increased a median 1.9% (IQR, 0.4% to 4.3%) in the states that did not expand Medicaid and 4.2% (IQR, 0.9% to 6.0%) in the states that did (Table 2). In the states that did not expand Medicaid, the Medicaid eligibility cutoff for parents decreased 6 percentage points of the FPL (IQR, −17.0 to 2.5), whereas in the states that did expand, the eligibility cutoff increased by 42.0 percentage points of the FPL (IQR, 5.0 to 81.0). Medicaid coverage for adults with 1 or more dependent children was found to increase (0.6 percentage points per year; 95% CI, 0.2 to 1.0) and was positively associated with Medicaid expansion status (1.6 percentage points higher for a given year; 95% CI, 0.1 to 3.0) (eTable 1 in the Supplement). In comparing the years 2013 and 2016, Medicaid expansion was found to be associated with a significant increase in percentage of FPL Medicaid eligibility, as would be expected (48.6 percentage points higher; 95% CI, 22.5 to 74.6).

**Table 2.** Net Change From 2013 to 2016 of Medicaid Coverage and Medicaid Eligibility Criteria for Adults With Dependent Children

Net Change	Medicaid Expansion, Median (IQR)	
	Present (n = 31)	Absent (n = 20)^a^
Medicaid coverage, %	4.2 (0.9 to 6.0)	1.9 (0.4 to 4.3)
Medicaid eligibility, % of FPL	42.0 (5.0 to 81.0)	−6.0 (−17.0 to 2.5)

Abbreviations: FPL, federal poverty level; IQR, interquartile range.

^a^ Louisiana began expansion in July 2016 and is included among the states that did not expand Medicaid.

**Figure 2.**
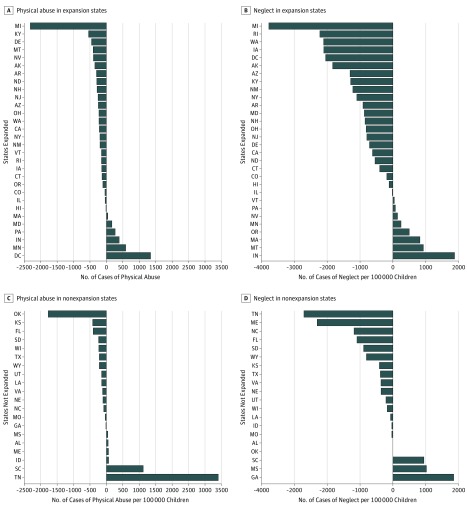
Change in the Physical Abuse and Neglect Rates Compared by State for 2013 vs 2016 Physical abuse and neglect rates are expressed as cases per 100 000 children younger than 6 years. Negative changes indicate the rate decreased from 2013 to 2016. Louisiana did not expand Medicaid until July 2016 and is classified with the nonexpansion states in this analysis. For West Virginia (not included), the change in physical abuse rates is thought to be an outlier not due to an actual change but to data unreliability concerns possibly related to a change in 2014 to the Child Protective Services intake system.^56^

In 2013, the baseline count of physical abuse cases was 176 591 in expansion states and 112 047 in nonexpansion states, or 1261 and 1138 per 100 000 children younger than 6 years, respectively. The baseline count of neglect cases was 646 463 in expansion states and 388 265 in nonexpansion states, or 4615 and 3944 per 100 000 respectively. From 2013 to 2016, many individual states in the Medicaid expansion and nonexpansion groups experienced decreased rates of physical abuse and neglect (Figure 2). When comparing the entire preexpansion and postexpansion periods, the states with Medicaid expansion as a whole saw the physical abuse rate decrease by 68 cases per 100 000 children younger than 6 years and the neglect rate decrease by 336 cases per 100 000 children younger than 6 years. In comparison, the group of states without Medicaid expansion had a decreased rate of physical abuse of 35 cases per 100 000 and an increased rate of neglect by 90 cases per 100 000 children younger than 6 years.

Table 3 shows the estimates for net change in the physical abuse and neglect rates after Medicaid expansion in the states that expanded Medicaid relative to the states that did not in any year. After Medicaid expansion, states that expanded Medicaid had fewer reported cases of neglect after adjusting for confounders (−422 per 100 000 children younger than 6 years; 95% CI, −753 to −91) compared with the change in the rate during that time in nonexpansion states, which had a baseline rate of 3944 per 100 000 children younger than 6 years in 2013 (Table 3). No statistically significant association of Medicaid expansion with physical abuse rates was found. Findings were similar when including West Virginia and excluding possible outlier state data (eTable 2 and eTable 3 in the Supplement). Neither Medicaid coverage itself nor Medicaid eligibility criteria were significantly associated with physical abuse or neglect rates, with or without adjusting for potential confounders (eTable 4 and eTable 5 in the Supplement). Based on these results, we estimated that had the nonexpansion states expanded Medicaid, a total of 124 981 (95% CI, 26 921-223 041) fewer cases of neglect would have been reported in the United States from 2014 through 2016, or a mean of 41 660 fewer cases per year.

**Table 3.** Changes in Annual Child Abuse and Neglect Rates in States That Expanded Medicaid Relative to States That Did Not^a^

Variable	Variable Estimate of Rate per 100 000 Children Aged <6 y for Each Study Year (95% CI)^b^	
	Physical Abuse	Neglect
Medicaid expansion	−57 (−213 to 99)	−422 (−753 to −91)^c^
Family policy	−101 (−661 to 459)	−1092 (−2282 to 98)
Unemployment rate	−62 (−125 to 1)	−85 (−219 to 49)
Families living below FPL	9 (−10 to 27)	31 (−9 to 71)
Teen births	5 (−21 to 31)	−8 (−63 to 48)
Childcare wait list	96 (−110 to 303)	153 (−287 to 592)

Abbreviation: FPL, federal poverty level.

^a^ The unadjusted change for physical abuse was −33 (95% CI, −186 to 119) per 100 000 children and for neglect was −427 (95% CI, −752 to −102) per 100 000 children (*P* < .05). The adjusted model controlled for the following state-level covariables: paid family leave policy, unemployment, teen birth rate, proportion of families living in poverty, and presence of a child care wait list.

^b^ Medicaid expansion indicates states with relative to those without this expansion. The analyses exclude West Virginia because its outcome data were found to be unreliable.

^c^ *P* < .05.

## Discussion

This study suggests an association between the ACA Medicaid expansion and reductions in the rate of screened-in reports made to CPS for concerns of neglect in children younger than 6 years. This association was seen even after controlling for other factors, including state-level policies and measures that might affect the reporting rate of child neglect and be associated with the status of Medicaid expansion in a state.

The results add to the small but growing body of evidence demonstrating a positive association between a social policy and child maltreatment outcomes.^15,16^ Neglect represents approximately three-quarters of reported child maltreatment cases in the United States.^57^ Despite being the most common form of child maltreatment, efforts to prevent neglect have proven difficult in part because of a lack of consensus regarding what constitutes neglect and how to best stratify risk for neglect.^57,58^ Our findings offer a promising avenue for future child neglect prevention research, especially as new states expand Medicaid.^17^

The absence of a significant association between Medicaid expansion and rates of reported physical abuse could be due to one of several possibilities. The first is that there may truly not be any association between Medicaid expansions and child physical abuse, suggesting that at least some of the pathways resulting in physical abuse are different from those resulting in neglect. An association may also exist between Medicaid expansion and physical abuse, but this association is seen only after more time has lapsed or only for a younger subset of children when compared with neglect. This possibility suggests that the association is delayed relative to that seen with neglect and was therefore not captured in the relatively short study period.

After state expansion occurred, some states saw much larger changes in eligibility and coverage than others. Therefore, we conducted further analyses to evaluate not only the association between Medicaid expansion and child maltreatment outcomes but also the associations between Medicaid expansion and these intermediate outcomes. We found that expanding Medicaid was associated with increases in the percentage of FPL eligibility cutoff as well as the proportion of parents covered by Medicaid. This finding is in line with those of prior research demonstrating that the expansion of Medicaid had the intended effect of increasing Medicaid coverage.^22,26,59^ Although Medicaid expansion was independently associated with the percentage of FPL eligibility cutoff and the proportion of parents covered by Medicaid and with neglect rates, no significant association was found between the former and the latter. This finding may suggest that the association between Medicaid expansion and neglect is complex and does not simply involve increased coverage. Another possibility is that a direct association exists between increased coverage and neglect but was not adequately detected in our analyses.

Medicaid coverage increased even in states that did not expand Medicaid. Other studies have attributed this finding to a “welcome-mat” effect among people who had been eligible for Medicaid before the expansions took place but only applied for Medicaid coverage after learning more about health care coverage through the national health care discussions.^59^ Despite this change, we found that the increase in Medicaid coverage for parents was significantly larger in the states that expanded Medicaid than in the states that did not.

### Limitations

This study had several limitations. Considering the Medicaid expansion as a binary exposure may not have fully captured some of the more complex changes that occurred within states. Medicaid expansion may be associated with other health care changes that improved patients’ ability to access care and services differently in different states, although they all opted to expand Medicaid. Medicaid delivery varies by state. Some of this variation is due to Medicaid waiver programs, which allow states to modify federal Medicaid rules to test different delivery and payment models.^60^ For example, as of December 7, 2018, there were 46 approved and 25 pending Section 1115 waivers pertaining to matters as varied as Medicaid work requirements and healthy behavior incentives.^61^ In addition to increasing Medicaid coverage, Medicaid expansion also involved states receiving federal funds to cover 100% of newly eligible Medicaid recipients.^62^ These additional funds may have allowed states to cover additional services affecting parents’ ability to provide for their children. Studying state-level measures of Medicaid delivery such as the number of health care professionals per capita accepting Medicaid, evaluating Medicaid waiver programs themselves, or looking at how states used the additional federal Medicaid funds may help identify specific pathways through which the association occurs between Medicaid expansion and decreased child neglect.

When considering Medicaid eligibility for parents based on income, considerable heterogeneity between states existed even before the ACA Medicaid expansion took place. We controlled for these baseline differences by conducting difference-in-difference analyses in which states were compared with themselves before and after Medicaid expansion. In addition, we were unable to capture and include state-level changes to child physical abuse and neglect policies and reporting laws that might have confounded our results. We attempted to control for these changes indirectly by controlling for other state-level variables that could have a bearing on them, although future research could evaluate the effect of these changes directly by collecting and including data on state-level policy changes over time. It has been previously demonstrated that only a subset of maltreated children are reported to CPS and therefore included in the NCANDS database used for this study.^63,64^ Including the entire population of maltreated children might alter the results by strengthening or weakening the association seen herein.

## Conclusions

This study found that ACA Medicaid expansion was associated with a reduction in the reported child neglect rate but with no significant change to the physical abuse rate. It appears from these findings that future studies should focus on assessing what the mechanism for this association is to determine whether expanding Medicaid prevents child neglect and whether further expansions could be designed to bolster this secondary association.

## Notes

Supplement. **eMethods.** Data Source and Main Outcome Models **eTable 1.** Change in Parental Medicaid Coverage and Medicaid Eligibility Criteria From 2013 to 2016 as a Function of Medicaid Expansion Status **eTable 2.** Changes in Annual Child Abuse and Neglect Rates per 100 000 Children Younger Than 6 Years in States That Expanded Medicaid Relative to States That Did Not Expand Medicaid Including West Virginia **eTable 3**. Changes in Annual Child Abuse and Neglect Rates per 100 000 Children Younger Than 6 Years in States That Expanded Medicaid Relative to States That Did Not Expand Medicaid Excluding Possible Outlier States **eTable 4.** Changes in Annual Child Abuse and Neglect Rates per 100 000 Children Younger Than 6 Years From 2013 to 2016 as a Function of Parental Medicaid Coverage **eTable 5.** Changes in Annual Child Abuse and Neglect Rates per 100 000 Children Younger Than 6 Years From 2013 to 2016 as a Function of Parental Medicaid Eligibility Criteria
