# Supplementary material for: Assessment of Rates of Child Maltreatment in States With Medicaid Expansion vs States Without Medicaid Expansion
Source: JAMA Netw Open. 2019 Jun 14;2(6):e195529. doi: 10.1001/jamanetworkopen.2019.5529 (PMC6575148; doi:10.1001/jamanetworkopen.2019.5529)
Supplement: Supplement. — eMethods. Data Source and Main Outcome Models eTable 1. Change in Parental Medicaid Coverage and Medicaid Eligibility Criteria From 2013 to 2016 as a Function of Medicaid Expansion Status eTable 2. Changes in Annual Child Abuse and Neglect Rates per 100 000 Children Younger Than 6 Years in States That Expanded Medicaid Relative to States That Did Not Expand Medicaid Including West Virginia eTable 3. Changes in Annual Child Abuse and Neglect Rates per 100 000 Children Younger Than 6 Years in States That Expanded Medicaid Relative to States That Did Not Expand Medicaid Excluding Possible Outlier States eTable 4. Changes in Annual Child Abuse and Neglect Rates per 100 000 Children Younger Than 6 Years From 2013 to 2016 as a Function of Parental Medicaid Coverage eTable 5. Changes in Annual Child Abuse and Neglect Rates per 100 000 Children Younger Than 6 Years From 2013 to 2016 as a Function of Parental Medicaid Eligibility Criteria [file jamanetwopen-2-e195529-s001.pdf]

Supplementary Online Content

Brown ECB, Garrison MM, Bao H, Qu P, Jenny C, Rowhani-Rahbar A. Assessment of rates of child maltreatment in states with Medicaid expansion vs states without Medicaid expansion. *JAMA Netw Open*. 2019;2(6):e195529. doi:10.1001/jamanetworkopen.2019.5529

eMethods. Data Source and Main Outcome Models

eTable 1. Change in Parental Medicaid Coverage and Medicaid Eligibility Criteria From 2013 to 2016 as a Function of Medicaid Expansion Status

eTable 2. Changes in Annual Child Abuse and Neglect Rates per 100 000 Children Younger Than 6 Years in States That Expanded Medicaid Relative to States That Did Not Expand Medicaid Including West Virginia

eTable 3. Changes in Annual Child Abuse and Neglect Rates per 100 000 Children Younger Than 6 Years in States That Expanded Medicaid Relative to States That Did Not Expand Medicaid Excluding Possible Outlier States

eTable 4. Changes in Annual Child Abuse and Neglect Rates per 100 000 Children Younger Than 6 Years From 2013 to 2016 as a Function of Parental Medicaid Coverage

eTable 5. Changes in Annual Child Abuse and Neglect Rates per 100 000 Children Younger Than 6 Years From 2013 to 2016 as a Function of Parental Medicaid Eligibility Criteria

This supplementary material has been provided by the authors to give readers additional information about their work.

©2019 Brown ECB et al. *JAMA Netw Open*.

eMethods. Data Source and Main Outcome Models

The analyses presented in this publication were based on data from the National Child Abuse and Neglect Data System (NCANDS) Child File, FFYs 2010v5, 2011v5, 2012v4, 2013v3, 2014v2, 2015v1, and 2016v1. These data were provided by the National Data Archive on Child Abuse and Neglect at Cornell University, and have been used with permission. The data were originally collected under the auspices of the Children's Bureau. Funding was provided by the Children's Bureau, Administration on Children, Youth and Families, Administration for Children and Families, U.S. Department of Health and Human Services. The collector of the original data, the funding agency, NDACAN, Cornell University, and the agents or employees of these institutions bear no responsibility for the analyses or interpretations presented here. The information and opinions expressed reflect solely the opinions of the authors.

Main outcome models

Unadjusted: $Y_{it} = \beta_0 + \beta_1 expansion_{it} + \gamma_i + \delta_t + \varepsilon_{it}$

Adjusted: $Y_{it} = \beta_0 + \beta_1 expansion_{it} + \beta_2 leave_{it} + \beta_3 unemp_{it} + \beta_4 teen_{it} + \beta_5 fpl_{it} + \beta_6 wait_{it} + \gamma_i + \delta_t + \varepsilon_{it}$

In these models, Y_{it} denotes the aggregated outcome rate calculated from individual-level data as the number of screened-in referrals for physical abuse or neglect per 100,000 children under the of 6 years for each state and each year, $expansion_{it}$ denotes whether or not a state had a Medicaid expansion at that year (0, 1, or a fraction for the states that expanded Medicaid in the middle of a year), and γ_i and δ_t respectively denote the state and year fixed effects. In addition, the vector of covariates for each state in each year included *leave* denoting paid family leave policy status, *unemp* denoting the unemployment proportion, *teen* denoting the teen pregnancy rate, *fpl* denoting the percentage of families with children under the age of 5 living below the FPL, *wait* denoting whether there was a child care wait list, and ε denoting the error term. Based on this model, β_1 is the effect of Medicaid expansion after accounting for covariates as well as state and year effects.

eTable 1. Change in Parental Medicaid Coverage and Medicaid Eligibility Criteria From 2013 to 2016 as a Function of Medicaid Expansion Status[a]

	Medicaid coverage	Medicaid eligibility
**Medicaid expansion estimate (95% CI[b])**	1.56 (0.10, 3.02)*	48.60 (22.50, 74.60)*

[a]Medicaid coverage is expressed as a percentage and Medicaid eligibility represents the percent of federal poverty level
[b]CI = confidence interval
*p-value < 0.05

©2019 Brown ECB et al. *JAMA Netw Open*.

eTable 2. Changes in Annual Child Abuse and Neglect Rates per 100 000 Children Younger Than 6 Years in States That Expanded Medicaid Relative to States That Did Not Expand Medicaid Including West Virginia[a]

Variable	Variable estimate (95% CI[b])	
	Physical abuse	Neglect
**Medicaid expansion**	140 (-103 , 382)	-399 (-724, -74)*
**Family policy**	91 (-789 , 971)	-1070 (-2250, 110)
**Unemployment rate**	76 (-20, 173)	-69 (-199, 61)
**Below FPL[c]**	46 (17, 74)*	35 (-3, 73)
**Teen births**	-41 (-81, 0)*	-13 (-67, 41)
**Waitlist**	29 (-296, 353)	145 (-291, 581)

[a]The unadjusted changes for physical abuse and neglect were 119 (-123, 360) and -407 (-728 , -87)* per 100,000 children respectively. The adjusted model presented above controlled for the following state-level covariables: paid family leave policy, unemployment, teen birth rate, proportion of families living in poverty, and presence of a child care wait list.
[b]CI = confidence interval
[c]FPL = federal poverty level
*p-value < 0.05

eTable 3. Changes in Annual Child Abuse and Neglect Rates per 100 000 Children Younger Than 6 Years in States That Expanded Medicaid Relative to States That Did Not Expand Medicaid Excluding Possible Outlier States[a]

Variable	Variable estimate (95% CI[b])	
	Physical abuse	Neglect
Medicaid expansion	26 (-78, 130)	-354 (-673, -35)*
Family policy	-170 (-535, 196)	-1303 (-2427, -180)*
Unemployment rate	-63 (-107, -20)*	-163 (-297, -29)*
Below FPL[c]	1 (-11, 14)	36 (-2, 74)
Teen births	-4 (-21, 14)	5 (-48, 58)
Waitlist	99 (-36, 233)	170 (-243, 583)

[a]The states excluded were Michigan, Oklahoma, Tennessee, and West Virginia. The unadjusted changes for physical abuse and neglect were 32 (-71, 136) and -347 (-667, -27)* per 100,000 children respectively. The adjusted model presented above controlled for the following state-level covariables: paid family leave policy, unemployment, teen birth rate, proportion of families living in poverty, and presence of a child care wait list.
[b]CI = confidence interval
[c]FPL = federal poverty level
*p-value < 0.05

©2019 Brown ECB et al. *JAMA Netw Open*.

eTable 4. Changes in Annual Child Abuse and Neglect Rates per 100 000 Children Younger Than 6 Years From 2013 to 2016 as a Function of Parental Medicaid Coverage[a]

Variable	Variable estimate (95% CI[b])			
	Model 1		Model 2	
	Physical abuse	Neglect	Physical abuse	Neglect
**Medicaid coverage**	-51 (-104, 2)	16 (-65, 97)	-46 (-100, 9)	25 (-58, 108)
**Family policy**	134 (-1470, 1737)	-1461 (-3913, 992)	167 (-1444, 1779)	-1398 (-3854, 1057)
**Unemployment rate**	-32 (-242, 178)	45 (-277, 366)	-50 (-264, 165)	12 (-315, 340)
**Below FPL[c]**	14 (-28, 56)	24 (-40, 88)	15 (-27, 58)	27 (-37, 92)
**Teen births**	-6 (-109, 97)	-11 (-169, 147)	3 (-103, 109)	5 (-156, 166)
**Waitlist**	230 (-650, 1110)	430 (-916, 1776)	231 (-653, 1114)	431 (-915, 1777)
**Medicaid expansion**			-196 (-663, 271)	-364 (-1076, 347)

[a]The unadjusted changes for physical abuse and neglect were -48 (-97, 1) and 16 (-60, 92) per 100,000 children respectively. Model 1 controlled for paid family leave, unemployment, teen birth rate, proportion of families with children under age 5 living below the federal poverty level, and presence of a child care wait list and model 2 controlled for the same covariates with the addition of Medicaid expansion.
The analyses above exclude West Virginia as its outcome data was found to be unreliable.
Medicaid coverage is expressed as a percentage and Medicaid eligibility represents the percent of federal poverty level
[b]CI = confidence interval
[c]FPL = federal poverty level

©2019 Brown ECB et al. *JAMA Netw Open*.

eTable 5. Changes in Annual Child Abuse and Neglect Rates per 100 000 Children Younger Than 6 Years From 2013 to 2016 as a Function of Parental Medicaid Eligibility Criteria[a]

Variable	Variable estimate (95% CI[b])			
	Model 1		Model 2	
	Physical abuse	Neglect	Physical abuse	Neglect
**Medicaid eligibility**	-4 (-8, 1)	-3 (-10, 4)	-3 (-8, 3)	-1 (-10, 7)
**Family policy**	-297 (-1951, 1356)	-1598 (-4075, 880)	-211 (-1920, 1498)	-1435 (-3991, 1121)
**Unemployment rate**	-56 (-270, 159)	35 (-286, 356)	-64 (-283, 155)	20 (-307, 348)
**Below FPL[c]**	11 (-32, 54)	20 (-44, 85)	13 (-31, 57)	24 (-43, 90)
**Teen births**	-2 (-107, 102)	-27 (-184, 130)	5 (-106, 116)	-13 (-178, 153)
**Waitlist**	191.92 (-702, 1086)	595 (-744, 1934)	175 (-731, 1080)	563 (-791, 1917)
**Medicaid expansion**			-132 (-686, 423)	-248 (-1077, 581)

[a]The unadjusted changes for physical abuse and neglect were -3 (-7, 1) and -1 (-8, 5) per 100,000 children respectively. Model 1 controlled for paid family leave, unemployment, teen birth rate, proportion of families with children under age 5 living below the federal poverty level, and presence of a child care wait list and model 2 controlled for the same covariates with the addition of Medicaid expansion.
The analyses above exclude West Virginia as its outcome data was found to be unreliable.
Medicaid coverage is expressed as a percentage and Medicaid eligibility represents the percent of federal poverty level
[b]CI = confidence interval
[c]FPL = federal poverty level

©2019 Brown ECB et al. *JAMA Netw Open*.
